# Effects of dietary supplementation of glucose oxidase, catalase, or both on reproductive performance, oxidative stress, fecal microflora and apoptosis in multiparous sows

**DOI:** 10.5713/ab.20.0839

**Published:** 2021-06-23

**Authors:** Xiaojiao Sun, Longguo Piao, Haifeng Jin, K. Margarette C. Nogoy, Junfang Zhang, Bin Sun, Yi Jin, Dong Hoon Lee, Seong-Ho Choi, Stephen B Smith, Xiangzi Li

**Affiliations:** 1 Department of Animal Science, Yanbian University, Yanji 133002, China; 2 CJ Cheiljedang feed (China) R&D center, Shenyang, Liaoning, 110000, China; 3 Department of Biosystems Engineering, Chungbuk National University, Cheongju 28644, Korea; 4 Department of Animal Science, Chungbuk National University, Cheongju 28644, Korea; 5 Department of Animal Science, Texas A&M University, College Station, TX 77843-2471, USA

**Keywords:** Apoptosis, Catalase, Fecal Microflora, Glucose Oxidase, Oxidative Stress, Sows

## Abstract

**Objective:**

The objective of this experiment was to investigate the effect of dietary glucose oxidase (GOD), catalase (CAT), or both supplementation on reproductive performance, oxidative stress, and apoptosis in sows.

**Methods:**

A total of 104 multiparous sows were randomly assigned to four groups (n = 26) with each group given a basal diet, basal diet plus GOD at 60 U/kg, basal diet plus CAT at 75 U/kg, and basal diet plus GOD at 60 U/kg and CAT at 75 U/kg. Sows were fed the experimental diets throughout gestation and lactation.

**Results:**

Dietary GOD supplementation increased average daily feed intake of sows and litter weight at weaning (p<0.05). Dietary CAT supplementation reduced the duration of parturition, stillbirth, and piglet mortality and increased growth performance of weaned piglets (p<0.05). Dietary GOD and CAT supplementation enhanced antioxidant enzyme activities and lessened oxidative stress product levels in plasma of sows and elevated antioxidant capacity of 14-day milk and plasma in weaned piglets (p<0.05). Dietary GOD supplementation increased fecal *Lactobacillus* counts and reduced *Escherichia coli* counts of sows (p<0.05). Compared with the basal diet, the GOD diet reduced fecal *Escherichia coli* counts of sows, but the addition of CAT did not reduce *Escherichia coli* counts in the GOD diet. Dietary GOD and CAT supplementation reduced the apoptosis rate of the liver, endometrium, and ovarian granulosa cells in sows (p<0.05). In the liver, uterus, and ovary of sows, the mRNA expression of caspase-3 and caspase-9 was downregulated by dietary GOD and CAT supplementation (p<0.05).

**Conclusion:**

Dietary GOD and CAT supplementation could improve the antioxidant capacity of sows and weaned piglets, and alleviate hepatic, ovarian and uterine apoptosis by weakening apoptosis-related gene expression. Glucose oxidase regulated fecal microflora of sows, but supplementation of CAT to GOD could weaken the inhibitory effect of GOD on fecal *Escherichia coli*.

## INTRODUCTION

Continuous genetic selection has led to modern sows producing larger litter sizes with each offspring being leaner and growing faster [1]. Consequently, the sows need to produce enough milk to meet the increasing demand for milk production in its fast-growing litter. With fetal growth accelerating and placental metabolism intensity rising in late pregnancy and lactation, the mater subsequently displays adaptive changes such as increasing uterine blood flow and rising glycerin, free fatty acid (FFA), and alanine contents in the blood [2], which means metabolism intensity of mater is improving. All of these lead to generating numerous endogenous reactive oxygen species (ROS). Excessive ROS reacts with proteins, lipids, DNA, and other organic macromolecules, and causes oxidative damage [3]. Besides, endogenous antioxidant enzyme activity is constantly declining with increasing parity [4], which cannot remove excess ROS timely. Therefore, it is hypothesized that supplementation of dietary antioxidants to multiparous sows could reduce oxidative stress, especially in late pregnancy and lactation.

Glucose oxidase (GOD) catalyzes β-D-glucose to produce hydrogen peroxide (H_2_O_2_) and β-D-gluconolactone by molecular oxygen, and β-D-gluconolactone spontaneously hydrolyzes to gluconic acid. Studies have shown that GOD is frequently utilized for improving the growth performance and immune function of animals due to gluconic acid and H_2_O_2_ produced by GOD in the gut [5,6]. Gluconic acid can improve intestinal health status and H_2_O_2_ inhibits the growth of harmful bacteria [7,8]. Wu et al [9] reported that GOD improved the growth performance of broilers mainly by enhancing intestinal digestive function and the abundance of beneficial bacterium. Meanwhile, catalase (CAT), decomposes H_2_O_2_ into water (H_2_O) and oxygen (O_2_), together with superoxide dismutase and glutathione peroxidase (GPx) constituting the enzyme defense system to scavenge ROS *in vivo*. For clinical analysis, CAT is associated with anti-aging, tumor pathogenesis, and imbalance of free radical metabolism [10]. *In vivo* results showed that continuous intravenous injection of exogenous CAT significantly enhanced plasma and lung CAT activities in rabbits [11]. Amini et al [12] reported that CAT (100 μg/mL) supplementation improved the activity of post-thawed sperm and reduced malondialdehyde (MDA) level in cock. Li et al [13,14] also indicated that the addition of CAT (2 g/kg diet) could increase CAT and total superoxide dismutase (T-SOD) activities in plasma and reduce the level of pro-inflammatory cytokines in the intestinal mucosa of piglets. Additionally, when GOD catalyzes the oxidation of glucose, H_2_O_2_ inhibits the catalysis of GOD [15], but CAT decomposes H_2_O_2_ to ensure a continuous and smooth reaction. This suggests that the interaction between GOD and CAT possibly has a better effect, which is of great guiding significance for the combined use of GOD and CAT as feed additives.

Effects of the addition of CAT on the oxidative capacity in piglets [13] and GOD on growth performance of piglets [5] have been reported, but there is no relevant literature available on the influence of combined dietary GOD and CAT on the reproductive performance and antioxidant capacity of sows. Therefore, this study was designed to evaluate how dietary GOD and CAT levels affected reproductive performance, antioxidant capacity, fecal microflora, and apoptosis of sows.

## MATERIALS AND METHODS

The experiment was conducted according to the Guideline for the Animal Care and Use Committee of Yanbian University.

### Experimental design

A total of 104 multiparous sows (parity 4.6±0.25, Yorkshire× Landrace) from CJ (Shenyang) Feed Co., Ltd. (Shenyang, Liaoning, China) were used in a 2×2 factorial treatment arrangement. The first-factorial level was GOD at 0 or 60 U/kg diet (Sunson Industry Group Co., Ltd., Beijing, China), and the second-factorial level was CAT at 0 or 75 U/kg diet (Shandong Chunxu Bio-Technology Co., Ltd., Jinan, China). Glucose oxidase and CAT were produced through fermentation by *Penicillium notatum*, and their activities were 200 U/g and 50 U/g, respectively. All sows were randomly assigned to receive 1 of 4 treatments (n = 26) and fed the experimental diets throughout gestation and lactation. The four treatments were as follows: basal diet, basal diet+75 U CAT/kg diet, basal diet+60 U GOD/kg diet, basal diet+60 U GOD/kg diet+75 U CAT/kg diet. This experiment was conducted from July to December 2018.

### Diets and management

The diets (Table 1) were formulated based on the recommendation of the NRC (2012). Sows were fed 2.0 kg/d from days 1 to 28 of gestation, 2.5 kg/d from days 28 to 84 of gestation, 3.0 kg/d from days 84 to 110 of gestation, and 1.8 kg/d from day 111 of gestation to farrowing. After farrowing, the diet amount was improved by adding 0.5 kg/d until *ad libitum* feeding during lactation. Piglets were weaned after 21 days of farrowing. Sows with similar body condition and parity (n = 5 per treatment) were randomly selected and slaughtered at day 21 of lactation to obtain liver, ovary, and uterus for gene expression and apoptosis analyses. The tissue samples were divided into two parts, one was frozen at −80°C, and the other was used for the paraffin section. The sows culled during the experiment were shown in Table 2. The back-fat thickness of sows was measured by ultrasound (Renco Corporation, Minneapolis, MN, USA) at P 2 position (60 mm off the midline at the 10th rib) during mating, on day 108 of gestation, farrowing day, and weaning. The number of piglets, litter weight, and average body weight (BW) were recorded after farrowing (day 1 of lactation) and after weaning (day 21 of lactation). Cross-fostering within the treatment group was implemented after 24 h of farrowing to attain 11±1 piglets per sow.

### Sampling

The blood samples (10 mL, n = 6 per treatment) from the sows were collected by jugular venipuncture at day 28, 80, and 108 of gestation (G28, G80, and G108, respectively), and day 1 and 14 of lactation (L1 and L14, respectively). The blood samples (5 mL, n = 6 per treatment) from the piglets were collected by jugular venipuncture at birth and weaning. Plasma samples anti-coagulated with sodium heparin were obtained by centrifugation at 3,000×g, 4°C for 15 min, and stored at −80°C. Colostrum and 14-d milk (n = 6 per treatment) were obtained by hand-expression according to the method of Tan et al [16]. The fecal samples of sows (n = 6 per treatment) were collected at G28, G108, and L14.

### Evaluation of antioxidant capacity

Antioxidant enzyme activities were analyzed in plasma and milk by commercially available kits (Nanjing Jiancheng Bioengineering Institute, Nanjing, China), including total antioxidant capacity (TAC), T-SOD, CAT, and GPx. The detection methods were reported by Meng et al [17]. Oxidative stress products including MDA, thiobarbituric acid reactive substances (TBARS), 8-hydroxy-deoxyguanosine (8-OHdG), and ROS in plasma or milk were measured by commercially available kits (Nanjing Jiancheng Bioengineering Institute, China), based on the methods of Zhao et al [18] and Tan et al [16].

### Quantification of microbial population

After bacterial DNA was extracted from feces, the standard curve was established through quantitative real-time polymerase chain reaction (PCR; Applied Biosystems, Foster City, CA, USA) with specific primers (Table 3) as the method of Tan et al [16]. The microbial DNA was diluted 10-fold for quantitative analysis. All values were expressed as colony-forming unit (cfu) per gram of wet feces (Log_10_ cfu/g).

### Terminal deoxyribonucleotide transferase-mediated dUTP nick-end labeling assay

The apoptosis of the liver, uterus, and ovary in sows was detected by the transferase-mediated dUTP nick-end labeling (TUNEL) apoptosis detection kit (KGA7072; Jiangsu KeyGEN Bio-tech Co., Ltd, Nanjing, China) as the instructions. Under the action of terminal deoxynucleotidyl transferase enzyme, fluorescein isothiocyanate-labeled 5′-triphosphate (dUTP) was inserted into the 3′-OH ends of the broken DNA in apoptotic cells. The chromogenic reaction was observed under a fluorescence microscope (Jingtong Instrument Co., Ltd, Suzhou, China). The nuclei showed blue fluorescence through 4′,6-diamidine-2-phenylindole staining, while apoptotic cells presented green fluorescence via TUNEL reaction mixture staining. Five fields (magnification, 400×) were randomly selected from each section, and the number of cells and apoptotic cells in each field were counted. The apoptotic rate was calculated.

### RNA extraction and real-time polymerase chain reaction

Total RNA was isolated from the liver, uterus, and ovary using TRIzol reagent (15596018; Invitrogen, Carlsbad, CA, USA). Complementary DNA synthesis was carried out with PrimeScript RT Reagent Kits (RR036A; Takara, Dalian, China) as per the instructions of the manufacturer. The specific primers in Table 3 were used to amplify target genes using 2 μL of the first-strand cDNA as the template in a 12.5 μL SYBR green-based quantitative real-time PCR reaction performed as follows: 95°C for 30 s, 40 cycles at 95°C for 5 s and 60°C for 30 s with a melting curve from 60°C to 95°C. β-Actin was served as a housekeeping gene to normalize gene expression levels in each sample. Relative expression was calculated using the 2^−ΔΔCt^ method.

### Statistical analysis

Data were analyzed by 2-way analysis of variance using the General Linear Model procedure of SPSS 20.0 for a 2×2 factorial design (IBM-SPSS Inc., Chicago, IL, USA). The model included the fixed effect of GOD level, CAT level and their interaction. Regarding piglet weight and litter weight at birth, the number of piglets at birth was used as a covariance. Piglet mortality was analyzed by Chi-square. For the sow and litter performance, an individual sow or litter was deemed to the experimental unit. All data were presented as means and standard error of mean. Statistical significance was set at p≤0.05 and tendency was declared at 0.05<p<0.10. When a significant interaction between GOD and CAT was found, Tukey’s multiple range test was used to further analyze the difference among the groups.

## RESULTS

### Reproductive performance of sows

For the performance of sows (Table 4), dietary GOD supplementation increased the average daily feed intake (ADFI) of sows during lactation (p<0.05), and dietary CAT supplementation shortened the duration of parturition (p<0.05). For the performance of piglets (Table 5), no significant interaction between GOD and CAT was found. Dietary GOD supplementation significantly elevated litter weight at weaning (p<0.05). Dietary CAT supplementation enhanced litter weight, BW, and ADG of weaned piglets, and reduced the number of stillbirth and pre-weaning mortality (p<0.05).

### Antioxidant status in plasma of sows

The effects of dietary GOD and CAT supplementation on antioxidant enzyme activities in plasma of sows were shown in Table 6. Dietary GOD supplementation increased T-SOD and GPx activities at L14 and TAC activity at G108 (p<0.05). Dietary CAT supplementation enhanced CAT activity at G108, L1 and L14, TAC activity at G108 and L1, T-SOD activity at L14, and GPx activity at L1 and L14 in plasma of sows (p< 0.05). The change of oxidative stress products in plasma was shown in Table 7. Dietary GOD supplementation decreased MDA level at L1 and L14 and 8-OHdG level at G108 and L1 (p<0.05). Meanwhile, dietary CAT supplementation reduced MDA and 8-OHdG levels at G108, L1, and L14 and ROS level at L1 (p<0.05). Interestingly, the levels of MDA and ROS revealed interactions between GOD and CAT at L1 and L14 (p<0.05). The addition of CAT reduced MDA level at L1 and L14 in the basal diet and reduced ROS level at L1 in the GOD diet. The TBARS level was not affected by dietary GOD and CAT supplementation.

### Antioxidant status in plasma of piglets

As shown in Table 8, dietary GOD supplementation significantly enhanced TAC and T-SOD activities and simultaneously reduced MDA level in plasma of weaned piglets (p<0.05). Dietary CAT supplementation enhanced TAC, T-SOD, CAT, and GPx activities and lessened MDA level in plasma of weaned piglets (p<0.05). Besides, dietary CAT supplementation elevated CAT activity in plasma of newborn piglets (p<0.05).

### Antioxidant status in colostrum and milk

The effects of dietary GOD and CAT supplementation on antioxidant capacity in milk were shown in Table 9. Dietary GOD supplementation significantly enhanced T-SOD and GPx activities in 14-d milk (p<0.05). Dietary CAT supplementation enhanced TAC, CAT, and GPx activities (p<0.05) and decreased MDA level (p<0.05) in 14-d milk. However, the antioxidant capacity of colostrum was unaffected by dietary GOD and CAT supplementation.

### Fecal microflora

Fecal Microflora of sows was shown in Table 10. There was a significant interaction between GOD and CAT on fecal *Escherichia coli* (*E. coli*) counts at G28, G108, and L14 (p<0.05). The GOD diet significantly reduced fecal *E. coli* counts (p< 0.05), but the addition of CAT did not inhibit *E. coli* counts in the GOD diet. Dietary GOD supplementation markedly reduced fecal *E. coli* counts and increased fecal *Lactobacillus* counts at G28, G108, and L14 (p<0.05).

### Apoptosis detected by transferase-mediated dUTP nick-end labeling assay

The apoptosis rate of the liver, endometrium, and ovarian granulosa cells in sows was detected by TUNEL *in situ* labeling at weaning (Table 11). Dietary GOD supplementation significantly reduced the apoptotic rate of the liver and ovarian granulosa cells (p<0.05). Accordingly, dietary CAT supplementation reduced the apoptosis rate of the liver, endometrium, and ovarian granulosa cells at day 21 of lactation (p<0.05).

### mRNA expression of apoptosis-related genes

In the liver (Table 12), dietary GOD supplementation markedly decreased caspase-3 and caspase-9 gene expression (p<0.05), while dietary CAT supplementation significantly reduced Bax/Bcl-2 ratio, caspase-3, and caspase-9 gene expression (p<0.05). In the ovary and uterus, dietary GOD and CAT supplementation lessened Bax/Bcl-2 ratio, caspase-3, and caspase-9 gene expression (p<0.05).

## DISCUSSION

The study hypothesized that dietary GOD and CAT supplementation could enhance reproductive performance and alleviate oxidative stress and apoptosis in sows. Tang et al [5] reported that dietary GOD (100 U/kg diet) supplementation in piglets had a greater ADG and lower feed conversion ratio. In this study, dietary GOD supplementation elevated feed intake of sows during lactation. This could be connected with gluconic acid, which was produced by GOD catalyzing glucose. In the obesity model, the most direct factors involved in insulin resistance are the increase of pro-inflammatory factors and FFA [19]. Gluconic acid produces volatile short-chain fatty acids (SCFA) through fermentation in the large intestine [7]. The increase of SCFA, especially acetic acid, is beneficial to decrease FFA concentration in the blood, which has been proved to improve insulin sensitivity [19]. It was also reported that improving insulin sensitivity during late pregnancy and early lactation could increase feed intake of sows during lactation [20]. Therefore, dietary GOD supplementation could elevate feed intake of sows during lactation, which may be related to the decrease of insulin resistance. In this study, dietary GOD supplementation improved litter weight at weaning, which could be associated with higher ADFI of sows. Sows having higher feed intake could produce enough milk to sustain the normal growth of piglets [21]. It is easy for sows to generate more weak and stillborn piglets if the duration of parturition is too long [22]. This study showed that dietary CAT supplementation decreased the duration of labor and stillbirth in sows. This is consistent with the previous study reporting that the stillborn rate of sows with a long duration of farrowing (>3 h) was twice that of sows with a short duration of farrowing (<3 h) [22]. Additionally, dietary CAT supplementation had lower weaned piglet mortality, which could be due to the lower oxidative stress level of sows. High intensity of the oxidative stress during lactation made sows upset, causing sows to move frequently after farrowing, and hence crushed one-week-old piglets [23]. Dietary CAT supplementation enhanced litter weight, BW, and ADG of piglets at weaning, suggesting that the growth performance of piglets from birth to weaning could be improved by milk. The quality of milk is affected by the health status of sows [23]. However, no significant interaction between GOD and CAT was observed on the reproductive performance of sows in this study.

With the rapid growth of fetus and milk yield, the demand for energy and oxygen levels of sows also continues to increase, which produces abundant ROS [3], resulting in DNA damage in sows and piglets [16]. Wang et al [24] observed that oxidative stress was markedly relevant to the reproductive performance of sows. Our study indicated that the antioxidant capacity of sows was improved by dietary GOD supplementation, which was demonstrated by the increase of TAC, T-SOD, and GPx activities and the decrease of MDA and 8-OHdG levels in plasma during late gestation and lactation (G108 and L14), alleviating the oxidative stress of sows. In this study, dietary CAT supplementation improved the antioxidant enzyme (TAC, T-SOD, CAT, and GPx) activities and decreased 8-OHdG and MDA concentrations in plasma of sows during late pregnancy and lactation (G108, L1, and L14). This is in accordance with the report showing that the addition of CAT elevated T-SOD and CAT activities and lessened MDA level in plasma of piglets [13]. Additionally, the interactions between GOD and CAT on plasma levels of MDA and ROS in sows during lactation showed that the ability of GOD to reduce the levels of the oxidative stress products was affected by dietary CAT level, and GOD combined with CAT could better alleviate the oxidative damage of lactating sows.

After farrowing, primiparous, old sows and their progeny may undergo severe oxidative stress [4]. The milk yield and reproductive performance of sows are reduced by the increase of oxidative stress level [18], which directly affects the health status of piglets. Nevertheless, antioxidants in milk can help piglets reduce oxidative stress and improve health status [25]. In the present study, dietary GOD supplementation enhanced the antioxidant enzyme (T-SOD and GPx) activities in 14-d milk. Meanwhile, dietary CAT supplementation improved the antioxidant enzyme (TAC, CAT, and GPx) activities and decreased MDA concentration in 14-d milk. These results indicate that dietary GOD and CAT supplementation partially elevates the antioxidant capacity of 14-d milk. This may be because the antioxidant status of milk is closely related to the levels of oxidative stress products in plasma of sows [23], and GOD and CAT improve the health status of sows, thus affecting the antioxidant capacity of milk. However, dietary GOD and CAT supplementation did not affect the antioxidant enzyme activities of colostrum, possibly because the improvement of antioxidant capacity of sows by GOD and CAT was not enough to influence antioxidant enzyme activities of colostrum. In this study, dietary GOD supplementation improved TAC and T-SOD activities and reduced MDA level in plasma of weaned piglets. Similarly, dietary CAT supplementation elevated antioxidant enzyme (TAC, T-SOD, CAT, and GPx) activities and reduced MDA level in plasma of weaned piglets. Additionally, milk was the only source of antioxidants and nutrients for piglets, because piglets were not fed creep feed or milk replacer during the trial period. These results show that dietary GOD and CAT supplementation can enhance the antioxidant status of milk. Limited studies reported the effects of dietary GOD and CAT supplementation on the antioxidant status of milk and suckling piglets, but some reports had indicated that the addition of antioxidants (i.e., selenium, vitamin E, and resveratrol) reduced the oxidative stress of piglets by improving the antioxidant capacity of sows and milk [17,26]. Our results were consistent with these reports and revealed that dietary GOD and CAT supplementation could improve the oxidative status of suckling piglets and milk by elevating the antioxidant capacity of lactating sows. Nevertheless, no interaction between GOD and CAT was found in any parameters of colostrum, milk, and piglets.

Reducing oxidative stress could benefit to improve the reproductive performance and gut health of sows. Wang et al [24] reported that gut microflora was different between the high and low reproductive performance of sows. In this study, we observed that dietary GOD supplementation during gestation and lactation reduced fecal *E. coli* counts, but increased fecal *Lactobacillus* counts. The reason may be that GOD regulates the host microflora by producing gluconic acid and H_2_O_2_ [5]. Biagi et al [7] observed that gluconic acid as a prebiotic effectively affected the variety of gut microflora. Short-chain fatty acids produced by gluconic acid fermentation, especially butyric acid, has antibacterial property, which inhibits the proliferation of *E. coli* and promotes the propagation of *Lactobacillus* and *Bifidobacterium* [27]. Moreover, H_2_O_2_ produced by GOD effectively inhibits and kills harmful microbe in the liquid whole egg [8]. Therefore, the results showed that dietary GOD supplementation had altered the gut microflora, which is beneficial to decrease ROS production, alleviating oxidative stress, and ultimately improved health status in sows. Nevertheless, during gestation and lactation, the counts of fecal *Bifidobacterium*, *Lactobacillus*, and *E. coli* were unaffected by dietary CAT supplementation, contradicting the result that dietary CAT enhanced the relative abundance of *Dialister* and *Bifidobacterium* in weaned piglets [14]. The contradiction may be due to the difference in the growth stage (sows versus weaned piglets). The interactions between GOD and CAT indicated that the inhibitory effect of GOD on *E. coli* varied with dietary CAT level throughout gestation and lactation, and the addition of CAT could weaken the inhibitory effect of GOD on *E. coli*. Although GOD produces H_2_O_2_, CAT can decompose H_2_O_2_ into H_2_O and O_2_, thus reducing the antimicrobial effect of H_2_O_2_ produced by GOD. These results are similar to the previous finding in the liquid whole egg [8].

Apoptosis is a genetically controlled and autonomously ordered cell death, which is different from necrotic cell death. Apoptosis plays a major role in maintaining normal metabolism and avoids inflammatory responses caused by cell death [28]. At present, it is believed that oxidative stress is closely related to cell apoptosis. Free radicals produced by metabolism or exogenous factors can directly induce apoptosis, which may be a ubiquitous mediator in the process of apoptosis [29]. Through TUNEL analysis, we found that dietary GOD supplementation reduced the apoptosis rate of liver and ovarian granulosa cells, while dietary CAT supplementation significantly decreased the apoptosis rate of the liver, endometrium, and ovarian granulosa cells. These results suggest that exogenous GOD and CAT can reduce the apoptosis of sows, and have certain protective effects on the liver, uterus and ovary during lactation.

A previous study reported that cell apoptosis involved the death receptor pathway and the mitochondrial pathway [30], which was primarily regulated by the caspase family, Bcl-2 family, and MAPK family. The caspase family is the promoter and executor of mammalian cell apoptosis [31]. Caspase-8 activates the death receptor pathway, while caspase-9 activates the mitochondrial pathway [30]. Activation of caspase-8 and caspase-9 further activates downstream caspase-3, which acts on specific apoptotic substrates and causes apoptosis [32]. In this study, dietary GOD and CAT supplementation down-regulated caspase-3 and caspase-9 gene expression in the liver, uterus and ovary of sows at weaning. This indicates that exogenous GOD and CAT suppress the apoptosis of the liver, uterus, and ovary by inhibiting caspase-9 activation, thus weakening caspase-3 activation. Additionally, previous studies had shown that the expression level of Bcl-2 and Bax directly influenced the regulation of apoptosis, and the Bcl-2/Bax ratio determined whether cells activated the inhibitory apoptosis mechanism or the accelerative apoptosis mechanism after stimulation [33,34]. In the study, although the gene expression of Bcl-2 and Bax related to mitochondrial apoptosis pathway in the liver and ovary of sows was not inhibited by dietary GOD and CAT supplementation, Bcl-2/Bax ratios in the liver, endometrium, and ovary were significantly decreased, inhibiting the occurrence of apoptosis, which further demonstrated that dietary GOD and CAT supplementation had a positive role in alleviating oxidative damage of lactating sows. However, no interaction between GOD and CAT was observed on cell apoptosis in the liver, ovary, and uterus.

In conclusion, dietary GOD and CAT supplementation elevated the reproductive performance of sows and improved the antioxidant status of sows and piglets. Dietary GOD supplementation regulated fecal microflora of sows, but CAT could reduce the inhibitory effect of GOD on *E. coli*. Dietary GOD and CAT supplementation alleviated the apoptosis of liver, ovary, and uterus by weakening caspase-3 and caspase-9 gene expression.

## Figures and Tables

**Table 1:** Composition of basal diets (as-fed basis)

Item	Gestation	Lactation
Ingredients (%)		
Corn	50.29	57.90
Sugar beet pulp	6.00	-
Wheat bran	20.00	3.00
Wheat flour	-	5.00
Corn germ meal	5.00	4.00
Dried distillers grains with solubles	6.00	-
Soybean meal, 43% crude protein	8.05	22.94
Fish meal	-	0.50
Soy oil	1.00	2.50
L-Lys-HCl, 78.8%	0.37	0.33
DL-Methionine, 98%	0.01	0.030
Limestone	1.50	1.50
Dicalcium phosphate	0.18	0.60
Salt	0.50	0.50
Choline chloride, 50%	0.10	0.20
Premix^1)^	1.00	1.00
Total	100.00	100.00
Calculated composition (%)		
Net energy (MJ/kg)	9.41	10.00
Crude protein	14.50	17.50
Crude fiber	6.01	4.41
Lysine	0.60	0.85
Methionine	0.21	0.27
Threonine	0.39	0.61
Calcium	0.85	1.00
Available phosphorus	0.32	0.36

1) Provided per kg diet: 15 mg Cu (CuSO_4_·5H_2_O), 90 mg Fe (FeSO_4_·H_2_O), 100 mg Zn (ZnSO_4_·H_2_O), 30 mg Mn (MnSO_4_·H_2_O), 0.5 mg I (CaI_2_O_6_), 0.3 mg Se (Na_2_SeO_3_), 7,000 IU vitamin A, 4,000 IU vitamin D_3_, 100 IU vitamin E, 4 mg vitamin K_3_, 4 mg Thiamin, 10 mg riboflavin, 7.5 mg vitamin B_6_, 0.06 mg vitamin B_12_, 45 mg D-pantothenate, 60 mg niacin, 0.5 mg biotin, 12 mg folic acid, xylanase 10,500 U, glucanase 600 U, cellulase 90 U, mannanase 1,200 U, phytase 3,000 FTU.

**Table 2:** Number of sows in different periods of experiment

Item	0 U/kg GOD		60 U/kg GOD	
	
	0 U/kg CAT	75 U/kg CAT	0 U/kg CAT	75 U/kg CAT
Breeding	26	26	26	26
Culled during gestation^1)^	2	1	2	1
Parturition	24	25	24	25
Culled during lactation^1)^	2	0	1	1
Weaning	22	25	23	24

GOD, glucose oxidase; CAT, catalase.

1) The culled sows refer to ill, metritis, reproductive failure, etc.

**Table 3:** Specific primers used for quantitative real-time polymerase chain reaction

Genes	Specific primers (5′-3′)	Product size (bp)	Accession number
*Lactobacillus* [16]	AGCAGTAGGGAATCTTCCA CACGCTACACATGGAG	341	NR_126253.1
*Bifidobacterium*	GATTCTGGCTCAGGATGAACGC CTGATAGGACGCGACCCCAT	230	MK_377258.1
*Escherichia coli* [16]	CATGCCGCGTGTATGAAGAA CGGGTAACGTCAATGAGCAAA	96	NR_024570.1
*Fas*	AGTTAAAGATTTCGTTCGG AGATACCAATTACGGAGC	117	NM_213839
*Bax*	TGACGGCAACTTCAACTGGG GCAGCCGATCTCGAAGGAA	143	XM_013998624.2
*Bcl-2*	TACCATCGGCGTAGTGC CCAAGGAGGTTCTGGAGTG	120	XM_021099593.1
*Caspase-3*	AACTCTAACTGGCAAACC GTCCCACTGTCCGTCTC	87	NM_214131.1
*Caspase-8*	TAGTGTAGCACGGAAGAAT GGTCCAAGTTTCGGTAG	179	XM_021074713.1
*Caspase-9*	CCCTTACCCTGCCTTACCT GGCTGCCGCATCCTTCA	102	XM_013998997.2
*β-actin*	GATTGGCATGGCTTTATTTG TCCATCCAACCGACTGCT	137	XM_003124280.3

**Table 4:** Effects of dietary GOD and CAT supplementation on performance of sows

Item	0 U/kg GOD		60 U/kg GOD		SEM	p-value		
		
	0 U/kg CAT	75 U/kg CAT	0 U/kg CAT	75 U/kg CAT		GOD	CAT	GOD×CAT
n^1)^	22	25	23	24				
Parity	4.77	4.84	4.35	4.50	0.267	0.156	0.682	0.874
Backfat of sows (mm)								
Breeding	14.43	14.00	14.33	14.31	0.479	0.830	0.643	0.664
Day108 of gestation	18.14	18.16	18.33	18.40	0.328	0.517	0.887	0.944
Gain	3.71	4.16	4.00	4.08	0.373	0.770	0.472	0.619
Parturition	18.43	18.36	18.39	18.23	0.319	0.789	0.714	0.888
Weaning	13.52	13.02	13.39	12.71	0.405	0.586	0.147	0.824
Loss	4.91	5.34	5.00	5.52	0.301	0.653	0.117	0.882
Lactation feed intake (kg/d)	6.58	6.43	6.67	6.91	0.111	0.013	0.711	0.090
Farrowing duration^2)^ (min)	202.50	180.40	185.65	170.71	8.341	0.115	0.029	0.669
WEI (d)	6.67	6.24	6.00	5.80	0.396	0.167	0.429	0.773

Data are means±standard error of the mean.

GOD, glucose oxidase; CAT, catalase; SEM, standard error of the mean; WEI, the weaning-to-estrus interval.

1) The number of sows for analysis.

2) Farrowing duration refers to the time interval between the birth of first piglet and the complete expulsion of placenta.

Statistical significance for main effects and interactions was set at p≤0.05 and tendency was declared at 0.05<p<0.10.

**Table 5:** Effect of dietary GOD and CAT supplementation on performance of piglets

Item	0 U/kg GOD		60 U/kg GOD		SEM	p-value		
		
	0 U/kg CAT	75 U/kg CAT	0 U/kg CAT	75 U/kg CAT		GOD	CAT	GOD×CAT
n	22	25	23	24				
Litter size (number/litter)								
Total born	11.82	12.20	12.13	12.13	0.456	0.795	0.681	0.672
Born alive	10.59	11.56	11.39	11.71	0.365	0.198	0.082	0.375
After cross-foster	10.73	10.72	10.87	10.79	0.169	0.527	0.801	0.835
Stillbirth	1.23	0.64	0.74	0.42	0.211	0.095	0.034	0.532
Mummy	0.36	0.20	0.26	0.13	0.124	0.473	0.228	0.911
Weaned piglets	9.68	10.20	10.17	10.38	0.192	0.086	0.064	0.411
Litter weight (kg)								
At birth^1)^	16.22	16.58	16.10	16.22	0.465	0.612	0.612	0.794
After cross-foster	16.64	16.49	16.75	16.70	0.304	0.600	0.747	0.874
At day 7	28.40	28.88	29.00	29.65	0.647	0.290	0.387	0.896
At day 21	54.84	59.76	59.27	62.82	1.674	0.028	0.013	0.683
Piglet mean BW (kg)								
At birth^1)^	1.46	1.48	1.44	1.45	0.041	0.498	0.763	0.843
After cross-foster	1.56	1.54	1.54	1.55	0.018	0.870	0.753	0.585
At day 7	2.69	2.72	2.73	2.78	0.045	0.289	0.319	0.886
At day 21	5.66	5.86	5.82	6.04	0.105	0.108	0.047	0.889
Piglet ADG (g/d)								
Day 1 to 7	161.56	169.22	169.01	175.80	5.105	0.173	0.161	0.932
Day 1 to 21	195.44	205.67	203.40	214.07	4.739	0.088	0.030	0.963
Piglet mortality^2)^ (%)								
At birth	9.59^a^	4.47^ab^	5.54^ab^	2.78^b^	-	-	-	0.007
At day 21	9.67^a^	4.83^b^	6.22^ab^	3.70^b^	-	-	-	0.037

Data are means±standard error of the mean.

GOD, glucose oxidase; CAT, catalase; SEM, standard error of the mean; BW, body weight; ADG, average daily gain.

1) Piglet number as a covariate.

2) Piglet mortality was analyzed by Chi-square.

Statistical significance for main effects and interactions was set at p≤0.05 and tendency was declared at 0.05<p<0.10.

a,b Means within a row with different superscripts differ (p≤0.05).

**Table 6:** Effects of dietary GOD and CAT supplementation on antioxidant enzyme activities in plasma of sows

Item	0 U/kg GOD		60 U/kg GOD		SEM	p-value		
		
	0 U/kg CAT	75 U/kg CAT	0 U/kg CAT	75 U/kg CAT		GOD	CAT	GOD×CAT
Day 28 of gestation								
TAC (U/mL)	3.18	3.38	3.23	3.60	0.180	0.461	0.135	0.640
T-SOD (U/mL)	126.58	132.20	128.64	133.42	4.357	0.712	0.250	0.924
CAT (U/mL)	4.51	4.44	4.10	4.74	0.223	0.809	0.223	0.132
GPx (U/mL)	221.48	224.98	223.45	226.08	3.320	0.650	0.369	0.897
Day 80 of gestation								
TAC (U/mL)	4.41	4.64	4.34	4.91	0.235	0.676	0.111	0.474
T-SOD (U/mL)	147.22	147.32	142.88	152.20	4.394	0.952	0.300	0.310
CAT (U/mL)	8.32	9.04	8.46	8.78	0.320	0.854	0.126	0.545
GPx (U/mL)	198.25	197.56	205.26	202.74	3.550	0.106	0.661	0.796
Day 108 of gestation								
TAC (U/mL)	5.08	5.60	5.38	6.31	0.206	0.026	0.003	0.320
T-SOD (U/mL)	138.38	144.48	140.66	147.38	4.005	0.527	0.129	0.939
CAT (U/mL)	7.20	8.08	7.79	8.31	0.278	0.158	0.022	0.524
GPx (U/mL)	217.97	221.92	219.29	221.81	3.141	0.850	0.318	0.823
Day 1 of lactation								
TAC (U/mL)	5.58	6.29	5.70	6.81	0.224	0.173	0.001	0.387
T-SOD (U/mL)	138.06	139.08	134.24	143.04	3.024	0.982	0.124	0.217
CAT (U/mL)	12.22	12.95	12.36	13.44	0.326	0.351	0.014	0.603
GPx (U/mL)	208.90	211.54	206.00	221.34	3.556	0.346	0.022	0.093
Day 14 of lactation								
TAC (U/mL)	4.22	4.27	4.17	4.64	0.205	0.446	0.222	0.320
T-SOD (U/mL)	90.48	97.18	96.22	105.00	3.064	0.042	0.022	0.739
CAT (U/mL)	5.23	5.52	5.09	6.31	0.283	0.265	0.017	0.121
GPx (U/mL)	187.12	194.68	190.22	209.12	4.112	0.049	0.005	0.187

Data are means±standard error of the mean (n = 6).

GOD, glucose oxidase; CAT, catalase; SEM, standard error of the mean; TAC, total antioxidant capacity; T-SOD, total superoxide dismutase; GPx, glutathione peroxidase.

Statistical significance for main effects and interactions was set at p≤0.05 and tendency was declared at 0.05<p<0.10.

**Table 7:** Effects of dietary GOD and CAT supplementation on oxidative stress products in plasma of sows

Item	0 U/kg GOD		60 U/kg GOD		SEM	p-value		
		
	0 U/kg CAT	75 U/kg CAT	0 U/kg CAT	75 U/kg CAT		GOD	CAT	GOD×CAT
Day 28 of gestation								
MDA (nmol/mL)	4.00	3.62	3.73	3.70	0.160	0.556	0.211	0.303
ROS (U/mL)	182.45	168.54	178.84	171.20	6.636	0.944	0.124	0.643
TBARS (nmol/mL)	65.77	60.85	63.25	62.36	2.080	0.810	0.181	0.347
8-OHdG (ng/mL)	19.01	17.73	18.05	18.13	0.554	0.615	0.292	0.235
Day 80 of gestation								
MDA (nmol/mL)	4.87	4.57	4.68	4.22	0.223	0.253	0.109	0.731
ROS (U/mL)	211.30	199.97	214.79	205.20	6.132	0.487	0.107	0.889
TBARS (nmol/mL)	81.25	78.62	79.69	75.86	2.253	0.352	0.171	0.794
8-OHdG (ng/mL)	28.26	27.22	27.33	26.52	0.566	0.170	0.122	0.836
Day 108 of gestation								
MDA (nmol/mL)	5.09	4.60	4.98	4.38	0.236	0.479	0.034	0.812
ROS (U/mL)	243.02	237.87	241.59	233.18	7.335	0.682	0.369	0.827
TBARS (nmol/mL)	123.55	116.17	120.59	113.18	4.304	0.499	0.105	0.998
8-OHdG (ng/mL)	39.17	37.86	38.13	36.35	0.511	0.024	0.008	0.647
Day 1 of lactation								
MDA (nmol/mL)	5.45^a^	4.37^b^	4.46^b^	4.28^b^	0.200	0.016	0.006	0.040
ROS (U/mL)	353.87^a^	341.00^ab^	367.90^a^	317.48^b^	8.806	0.597	0.002	0.049
TBARS (nmol/mL)	164.22	156.22	159.84	154.76	4.040	0.481	0.125	0.722
8-OHdG (ng/mL)	42.94	40.21	41.38	39.19	0.550	0.032	<0.001	0.633
Day 14 of lactation								
MDA (nmol/mL)	4.43^a^	3.44^b^	3.60^b^	3.29^b^	0.156	0.006	0.001	0.046
ROS (U/mL)	226.25	222.91	230.60	213.79	6.235	0.707	0.126	0.296
TBARS (nmol/mL)	73.16	71.74	71.69	68.96	2.865	0.469	0.479	0.823
8-OHdG (ng/mL)	33.58	32.18	32.57	31.64	0.509	0.149	0.036	0.648

Data are means±standard error of the mean (n = 6).

GOD, glucose oxidase; CAT, catalase; SEM, standard error of the mean; MDA, malondialdehyde; ROS, reactive oxygen species; TBARS, thiobarbituric acid reactive substances; 8-OHdG, 8-hydroxy-deoxyguanosine.

Statistical significance for main effects and interactions was set at p≤0.05 and tendency was declared at 0.05<p<0.10.

a,b Means within a row with different superscripts differ (p≤0.05).

**Table 8:** Effects of dietary GOD and CAT supplementation on antioxidant status in plasma of piglets

Item	0 U/kg GOD		60 U/kg GOD		SEM	p-value		
		
	0 U/kg CAT	75 U/kg CAT	0 U/kg CAT	75 U/kg CAT		GOD	CAT	GOD×CAT
At birth								
TAC (U/mL)	7.75	8.19	8.04	8.31	0.218	0.352	0.119	0.702
T-SOD (U/mL)	117.56	120.04	122.22	124.40	2.826	0.130	0.422	0.958
CAT (U/mL)	2.87	3.12	2.94	3.32	0.145	0.364	0.042	0.679
GPx (U/mL)	144.90	151.10	149.10	157.54	4.41	0.342	0.171	0.637
MDA (nmol/mL)	3.08	2.99	2.92	2.74	0.164	0.239	0.410	0.778
At weaning								
TAC (U/mL)	8.24	8.73	8.76	9.40	0.261	0.038	0.046	0.786
T-SOD (U/mL)	131.54	139.42	141.26	146.68	3.041	0.013	0.044	0.691
CAT (U/mL)	3.12	3.59	3.35	3.75	0.189	0.309	0.035	0.864
GPx (U/mL)	162.00	170.90	167.76	179.54	4.795	0.153	0.047	0.768
MDA (nmol/mL)	3.48	2.77	2.71	2.52	0.204	0.024	0.044	0.218

Data are means±standard error of the mean (n = 6).

GOD, glucose oxidase; CAT, catalase; SEM, standard error of the mean; TAC, total antioxidant capacity; T-SOD, total superoxide dismutase; GPx, glutathione peroxidase; MDA, malondialdehyde.

Statistical significance for main effects and interactions was set at p≤0.05 and tendency was declared at 0.05<p<0.10.

**Table 9:** Effects of dietary GOD and CAT supplementation on the antioxidant status of milk

Item	0 U/kg GOD		60 U/kg GOD		SEM	p-value		
		
	0 U/kg CAT	75 U/kg CAT	0 U/kg CAT	75 U/kg CAT		GOD	CAT	GOD×CAT
Colostrum								
TAC (U/mL)	26.10	28.79	27.95	29.55	1.273	0.320	0.111	0.675
T-SOD (U/mL)	265.52	270.52	269.84	277.90	3.791	0.142	0.104	0.692
CAT (U/mL)	7.66	8.24	8.07	8.37	0.263	0.315	0.113	0.589
GPx (U/mL)	120.90	124.24	126.88	130.42	4.134	0.161	0.418	0.981
MDA (nmol/mL)	6.18	6.15	6.28	5.97	0.263	0.861	0.530	0.605
14-day milk								
TAC (U/mL)	21.95	24.94	23.36	24.54	0.699	0.481	0.009	0.216
T-SOD (U/mL)	221.42	229.96	232.86	238.20	3.485	0.012	0.064	0.652
CAT (U/mL)	4.99	5.65	5.38	6.08	0.205	0.059	0.004	0.912
GPx (U/mL)	90.22	99.10	98.42	102.22	2.579	0.043	0.026	0.339
MDA (nmol/mL)	5.79	5.26	5.54	4.83	0.217	0.135	0.012	0.674

Data are means±standard error of the mean (n = 6).

GOD, glucose oxidase; CAT, catalase; SEM, standard error of the mean; TAC, total antioxidant capacity; T-SOD, total superoxide dismutase; GPx, glutathione peroxidase; MDA, malondialdehyde.

Statistical significance for main effects and interactions was set at p≤0.05 and tendency was declared at 0.05<p<0.10.

**Table 10:** Effects of dietary GOD and CAT supplementation on fecal bacterial counts in sows

Item	0 U/kg GOD		60 U/kg GOD		SEM	p-value		
		
	0 U/kg CAT	75 U/kg CAT	0 U/kg CAT	75 U/kg CAT		GOD	CAT	GOD×CAT
*Bifidobacterium* (Log_10_cfu/g)								
G28	9.34	9.63	9.76	9.97	0.238	0.127	0.311	0.866
G108	9.13	9.42	9.59	9.54	0.162	0.091	0.466	0.312
L14	8.75	8.84	9.11	9.20	0.192	0.079	0.639	0.992
*Lactobacillus* (Log_10_cfu/g)								
G28	10.88	11.06	11.58	11.83	0.140	<0.001	0.138	0.822
G108	11.04	11.26	11.92	12.04	0.147	<0.001	0.261	0.743
L14	10.72	11.08	11.52	11.71	0.165	0.001	0.111	0.623
*Escherichia coli* (Log_10_cfu/g)								
G28	12.31^a^	12.01^ab^	11.51^b^	11.90^ab^	0.137	0.004	0.791	0.023
G108	12.09^a^	11.82^a^	11.34^b^	11.62^ab^	0.115	0.001	0.973	0.030
L14	12.48^a^	12.08^ab^	11.62^b^	11.97^ab^	0.136	0.002	0.856	0.014

Data are means±standard error of the mean (n = 6).

GOD, glucose oxidase; CAT, catalase; SEM, standard error of the mean; cfu, colony-forming unit.

Statistical significance for main effects and interactions was set at p≤0.05 and tendency was declared at 0.05<p<0.10.

a,b Means within a row with different superscripts differ (p≤0.05).

**Table 11:** Effect of dietary GOD and CAT supplementation on the apoptosis rate of the liver, endometrium, and ovarian granulosa cells in sows at weaning

Item	0 U/kg GOD		60 U/kg GOD		SEM	p-value		
		
	0 U/kg CAT	75 U/kg CAT	0 U/kg CAT	75 U/kg CAT		GOD	CAT	GOD×CAT
Liver (%)	12.06	9.70	10.02	8.65	0.720	0.048	0.020	0.501
Endometrium (%)	9.98	7.98	8.60	7.91	0.437	0.115	0.007	0.153
Ovarian granulosa cells (%)	15.36	10.89	12.26	9.80	0.950	0.043	0.002	0.307

Data are means±standard error of the mean (n = 5).

GOD, glucose oxidase; CAT, catalase; SEM, standard error of the mean.

Statistical significance for main effects and interactions was set at p≤0.05 and tendency was declared at 0.05<p<0.10.

**Table 12:** Effects of dietary GOD and CAT supplementation on apoptosis-related gene expression of the liver, ovary, and uterus in sows at weaning

Item	0 U/kg GOD		60 U/kg GOD		SEM	p-value		
		
	0 U/kg CAT	75 U/kg CAT	0 U/kg CAT	75 U/kg CAT		GOD	CAT	GOD×CAT
Liver								
Fas	1.000	0.936	0.955	0.909	0.033	0.278	0.108	0.783
Bax	1.000	0.890	0.922	0.918	0.040	0.518	0.163	0.194
Bcl-2	1.000	1.133	1.094	1.147	0.045	0.255	0.057	0.402
Bax/Bcl-2 ratio	1.000	0.791	0.849	0.812	0.053	0.232	0.033	0.120
Caspase-3	1.000	0.861	0.891	0.832	0.027	0.020	0.002	0.147
Caspase-8	1.000	0.895	0.920	0.907	0.052	0.515	0.268	0.389
Caspase-9	1.000	0.872	0.884	0.816	0.040	0.048	0.028	0.470
Ovary								
Fas	1.000	0.943	0.961	0.965	0.032	0.783	0.410	0.340
Bax	1.000	0.940	0.954	0.886	0.045	0.277	0.170	0.943
Bcl-2	1.000	1.124	1.125	1.106	0.044	0.250	0.262	0.128
Bax/Bcl-2 ratio	1.000	0.836	0.846	0.813	0.038	0.034	0.021	0.104
Caspase-3	1.000	0.906	0.903	0.837	0.035	0.030	0.037	0.684
Caspase-8	1.000	1.008	0.981	0.955	0.035	0.310	0.792	0.663
Caspase-9	1.000	0.840	0.863	0.804	0.031	0.012	0.002	0.114
Uterus								
Fas	1.000	0.926	0.948	0.958	0.038	0.777	0.400	0.277
Bax	1.000	0.939	0.947	0.903	0.037	0.235	0.167	0.803
Bcl-2	1.000	1.222	1.147	1.205	0.059	0.293	0.032	0.188
Bax/Bcl-2 ratio	1.000	0.779	0.828	0.754	0.038	0.018	0.001	0.067
Caspase-3	1.000	0.883	0.910	0.849	0.029	0.047	0.007	0.343
Caspase-8	1.000	0.934	0.992	0.977	0.033	0.616	0.233	0.442
Caspase-9	1.000	0.867	0.882	0.804	0.037	0.025	0.011	0.450

Data are means±standard error of the mean (n = 5).

GOD, glucose oxidase; CAT, catalase; SEM, standard error of the mean.

Statistical significance for main effects and interactions was set at p≤0.05 and tendency was declared at 0.05<p<0.10.
